# Screening of Deletion Variants within the Goat *PRDM6* Gene and Its Effects on Growth Traits

**DOI:** 10.3390/ani10020208

**Published:** 2020-01-27

**Authors:** Zhen Wang, Congliang Wang, Yongni Guo, Shuaishuai She, Baojing Wang, Yuru Jiang, Yangyang Bai, Xiaoyue Song, Longping Li, Lei Shi, Lei Qu, Xianyong Lan, Haijing Zhu

**Affiliations:** 1 Shaanxi Provincial Engineering and Technology Research Center of Cashmere Goats, Yulin University, Yulin 719000, Shaanxi, China; wangzhenid@126.com (Z.W.); wcl15596064203@126.com (C.W.); guoyongni@126.com (Y.G.); sheshuaishuai0513@126.com (S.S.); wangbaoj@126.com (B.W.); jiangyuru@126.com (Y.J.); bai345@126.com (Y.B.); songxiaoyue@yulinu.edu.cn (X.S.); llp_315@163.com (L.L.); shilei_ylxy@126.com (L.S.); ylqulei@126.com (L.Q.); 2 Life Science Research Center, Yulin University, Yulin 719000, Shaanxi, China; 3 Key Laboratory of Animal Genetics, Breeding and Reproduction of Shaanxi Province, College of Animal Science and Technology, Northwest A&F University, Yangling 712100, Shaanxi, China

**Keywords:** goats, *PRDM6*, deletion, growth traits, correlation analysis

## Abstract

**Simple Summary:**

Genome-wide association studies found that the PR/SET Domain 6 (*PRDM6*) gene mutation was associated with bone development, bone density, and body mass index. This study found a 12 bp deletion variation within the *PRDM6* gene in Shaanbei white cashmere goats in a large sample size (*n* = 1044). This variation polymorphism was associated with multiple goat growth traits in the yearling period, including heart girth (*p* = 0.027), cannon circumference (*p* = 0.008), chest depth (*p* = 2.10 × 10^−5^), chest width (*p* = 0.004), body height (*p* = 0.032), body length (*p* = 0.044), and hip-width (*p* = 0.014). The effects of the 12 bp variation were found to make no difference on adult goat growth traits. Taken together, these results demonstrate that the 12 bp deletion variant plays an important role in the early growth and development of goats and could be considered as a useful and effective molecular marker for goat breeding selection in the growth stage.

**Abstract:**

By genome-wide association studies, the *PRDM6* gene has been shown to affect multiple, apparently unrelated inherited traits, including bone density and body mass index. Therefore, it is considered a potentially pleiotropic gene. In this study, we identified a 12 bp deletion variant (NC_030814.1:rs651603667, g: 79985625-79985636delTTGACTGATCCA) within the *PRDM6* gene in a large sample (SBWC goats; *n* = 1044). All goat samples were collected in Shaanxi province in July 2018. The frequency of the wt allele was higher than the frequency of the del allele, and this mutation polymorphism confirmed to be consistent with the Hardy–Weinberg equilibrium (*p* > 0.05). Further results showed that in a group of goats in the yearling period (18 months old, *n* = 567), this deletion variant of the *PRDM6* gene was associated with heart girth (*p* = 0.027), cannon circumference (*p* = 0.008), chest depth (*p* = 2.10 × 10^−5^), chest width (*p* = 0.004), body height (*p* = 0.032), body length (*p* = 0.044) and hip-width (*p* = 0.014). For adult SBWC goats (36 months old, *n* = 477), the effects of the 12 bp variation on growth-related traits were found to make no difference. These findings show that the 12 bp deletion within the goat *PRDM6* gene plays an important role in the early growth and development of goats. Using the 12 bp mutation, breeders can quickly and effectively select excellent individual goats at an early stage.

## 1. Introduction

China is rich in livestock resources and has a wide variety of animal species, which means that the development and utilization of multiple livestock species deserve our attention. Livestock serves the human population in many ways, in particular, by providing protein-rich meat. Goat meat, with its rich nutrition favored by meat consumers, plays a vital role in daily diets [1,2]. For instance, goat meat contains low levels of cholesterol and intramuscular fat [3]. However, in many countries, and in China, in particular, poor production performance and quality still represent a bottleneck that restricts the advancement of goat meat production [4]. Hence, finding useful and practical measures to improve goat growth traits is urgent. As a meat-fleece hybrid goat breed, Shaanbei white cashmere (SBWC) goats have excellent fluff producing properties. However, they still have the disadvantages of small body size, slow growth and a long raise period; therefore, it is meaningful to pay attention to the breeding of growth traits in SBWC goats. By traditional methods, the breeding process in the goat industry generally requires a lot of time, which is inefficient and costly. With the development of modern breeding technology and breeding industry, we wanted to explore a useful and efficient way to promote goat growth traits and enhance economic performance. Many studies have confirmed that polymorphisms of some candidate genes could be associated with economic traits in livestock [5,6,7]. Van Laere found that a G to A nucleotide mutation appeared in intron 3 of porcine *IGF2*, which abolishes BED-type containing 6 repressor (a kind of transcription factor) binding and affected pig meat production [8]. Therefore, the genetic selection of economic traits in goats has been widely studied and reported and is of particular significance [9,10,11]. 

Recently, using genome-wide association studies (GWASs), the *PRDM6* gene has been shown to affect multiple, apparently unrelated inherited traits, including bone density and body mass index; therefore, it was regarded as a potentially pleiotropic gene [12,13]. The formation of the skeleton is part of the early development of an organism [14,15]. Hu et al. found that the *PRDM6* mutation was associated with bone development, bone density, and body mass index [12]. We hypothesized that the *PRDM6* gene might play an essential role in early growth stages and might affect goat growth and development. However, whether the *PRDM6* gene mutation will influence the growth traits and body shape of goats has still not been reported and is not yet clear. In this study, we investigated whether *PRDM6* is related to goat growth in the yearling (18 months old) and adult (36 months old) periods, to provide some scientific basis for the efficient and rapid development of the goat industry.

## 2. Materials and Methods 

### 2.1. Ethics Statement

The Faculty Animal Policy and Welfare Committee of Northwest A & F University under contract (NWAFU-314020038) approved this experiment, and the experimental process complied with protocols of international guide for the ethical use of animals in research.

### 2.2. Goat Ear Tissues and Collection of Growth Trait Data 

A total of 1044 healthy female SBWC goats, including 567 yearlings (18 months old) and 477 adults (36 months old), were selected randomly to make sure the individuals were not related. Yearling means that goats have not reached body maturity and have not been mated, and adult means that these goats have reached body maturity and have been mated. They were raised on a Shaanbei white cashmere goat farm in Shaanxi Province. Their feeding and living conditions were the same. Growth trait data of SBWCs were measured according to our previous methods [16,17], including heart girth (HG), hip-width (HW), cannon circumference (CC), height at hip cross (HHC), chest depth (CD), chest width (CW), body height (BH), body length (BL) and body weight (BWT). All growth trait data were verified by the technical agricultural station [11]. All ear tissues were collected in July 2018 and immediately soaked in 75% alcohol and taken back to the lab and frozen at −80 °C. Genomic DNA was isolated using the high salt-extraction method from ear tissues [18]. The concentrations of 1044 samples were measured by a Nanodrop 2000 Spectrophotometer, and were diluted to 20 ng/μL and frozen at −40 °C for further experiments [19,20].

### 2.3. Primer Design and PCR Amplification

From the Ensembl database (https://asia.ensembl.org/index.html), we found goat *PRDM6* gene variations. According to the variant table of the *PRDM6* gene, two putative deletion mutations in upstream and intron 1 of the *PRDM6* gene had not been identified before. The two pairs of primers were designed by Primer-Blast of the NCBI website (https://www.ncbi.nlm.nih.gov/tools/primer-blast) based on the goat *PRDM6* sequence (NC_030814.1). The PCR reaction contained 1.2 ng genomic DNA using the touchdown PCR procedure. PCR amplification was performed with an initial denaturation at 95 °C for 5 min, followed by 18 cycles at 94 °C for 30 s, 68 °C to 50 °C for 30 s and 72 °C for 12 s, then 34 cycles at 94 °C for 30 s, 50 °C for 30 s and 72 °C for 12 s, with a final extension at 72 °C for 10 min. PCR products were detected by Sanger sequencing and electrophoresis in agarose gel at 3.5% concentration [21]. 

### 2.4. Statistical Analyses

Associations of the 12 bp variation locus within the *PRDM6* gene with the growth traits of all 1044 individuals were determined using a linear model to show the effects of different parameters on the growth traits of goats. There were influences of different parameters on growth traits: S_ij_ = µ + G_i_ + Y_j_ + e_ij_. S_ij_ represents the values of growth traits, µ is the mean, G_i_ is the genotype effect, Y_j_ represents effects of age, and e_ij_ is random error [22]. One-way analysis of variance was performed to analyze the correlations between genotypes and growth traits using the SPSS 23.0 software [11]. The analysis results are presented in the form of mean ± standard error (SE). Homozygosity (Ho), heterozygosity (He), and the polymorphism information content (PIC) were calculated according to a previous method [23]. Ho, He, PIC, and the Hardy–Weinberg equilibrium (HWE) were performed using the SHEsis website (http://analysis.bio-x.cn) [24,25].

## 3. Results

### 3.1. Identification of Insertion/Deletion Polymorphisms within the PRDM6 Gene

**Figure 1:**
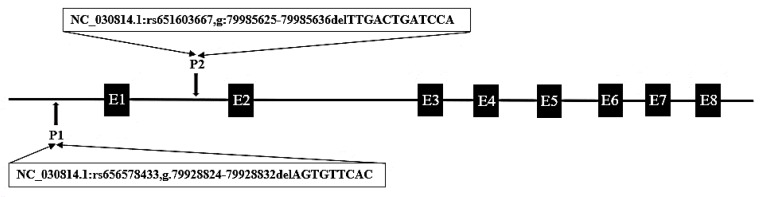
Mode pattern of goat *PRDM6* gene variations position. The black arrows represent the mutation positions; the black boxes represent the number of exons of the goat *PRDM6*.

**Figure 2:**
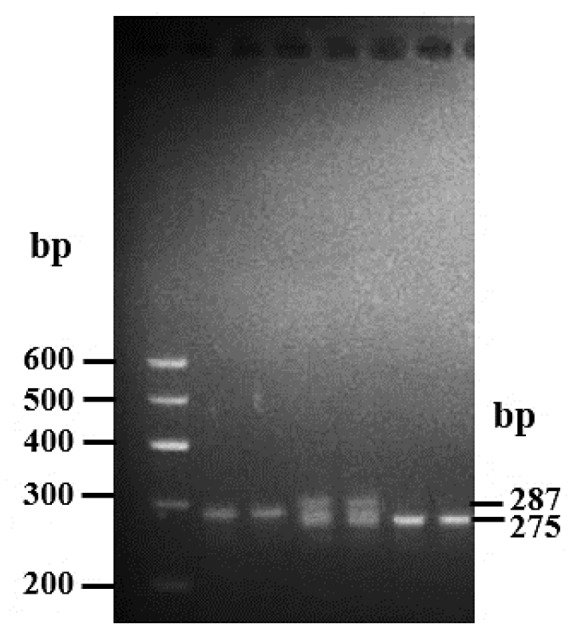
Electrophoresis pattern of the 12 bp deletion locus within the goat *PRDM6* gene. Results with marker, wt/wt, wt/wt, wt/del, wt/del, del/del, del/del display. Wildtype, wt/wt; heterozygote genotype, wt/del, homozygote deletion genotype, del/del.

**Figure 3:**
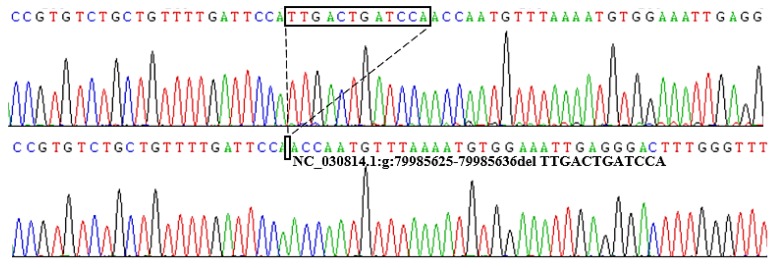
Sequencing diagram of the 12 bp deletion locus within the goat *PRDM6* gene. The sequence within the black border shows the difference in the sequence fragment.

From the Ensembl database (https://asia.ensembl.org/index.html), two putative deletion variants were detected in goat *PRDM6* (rs656578433; rs651603667), and their specific positions are shown in Figure 1. The 9 bp deletion mutation (NC_030814.1:rs656578433, g.79928824-79928832delAGTGTTCAC) was located upstream of the *PRDM6* gene, and the 12 bp deletion variation (NC_030814.1:rs651603667, g: 79985625-79985636delTTGACTGATCCA) was located in intron 1 of *PRDM6*. Compared with the wild sequence, 9 bp and 12 bp sequences, respectively, were missing at these two sites. We designed two pairs of specific primers to amplify these two deletion mutations. The results show that only the 12 bp deletion locus was polymorphic in the 1044 SBWC goats. The 12 bp deletion within *PRDM6* was detected by P2 (Table 1). The results of agarose gel electrophoresis and sequencing diagrams used to identify the genotypes of the 12 bp variations within *PRDM6* are presented in Figure 2 and Figure 3. The results show that the 12 bp mutation locus of the *PRDM6* gene was polymorphic and could be detected by agarose gel electrophoresis and Sanger sequencing.

### 3.2. Genotyping and Genetic Parameters of Genetic Variations of the PRDM6 Gene

The mean values and sample sizes of goat growth traits in yearling and adult goats are represented in Table 2. The results of electrophoresis show that the 12 bp deletion variation within the *PRDM6* gene contained three genotypes, including homozygous wildtype (wt/wt genotype, 287 bp), heterozygote genotype (wt/del genotype, 287 bp and 275 bp), and homozygous deletion genotype (del/del genotype, 275 bp) (Figure 2). According to the results of the three genotypes, sequence variation frequencies and population indexes of the 12 bp mutation of *PRDM6* in yearling and adult goats were calculated. In both populations, the genotype distributions in the 12 bp variations within the *PRDM6* gene were consistent with the HWE (*p* > 0.05) and were of medium genetic diversity (0.25 < PIC < 0.5) (Table 3).

### 3.3. Correlations of the 12 bp Deletion within PRDM6 with Goat Growth Traits

**Figure 4:**
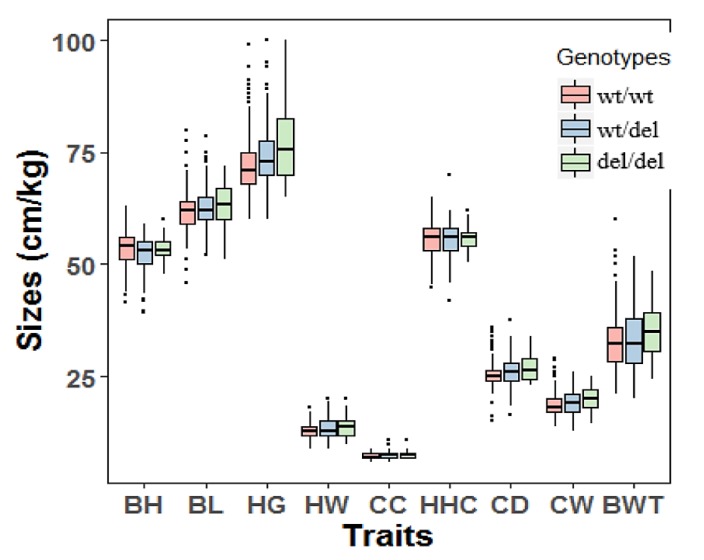
The associations between the 12 bp deletion of *PRDM6* and body measurement traits of yearling Shaanbei cashmere goats. Wildtype, wt/wt; heterozygote genotype, wt/del, homozygote deletion genotype, del/del. Body height (cm), BH; body length (cm), BL; heart girth (cm), HG; hip-width (cm), HW; cannon circumference (cm), CC; height at hip cross (cm), HHC; chest depth (cm), CD; chest width (cm), CW; body weight (kg), BWT.

**Figure 5:**
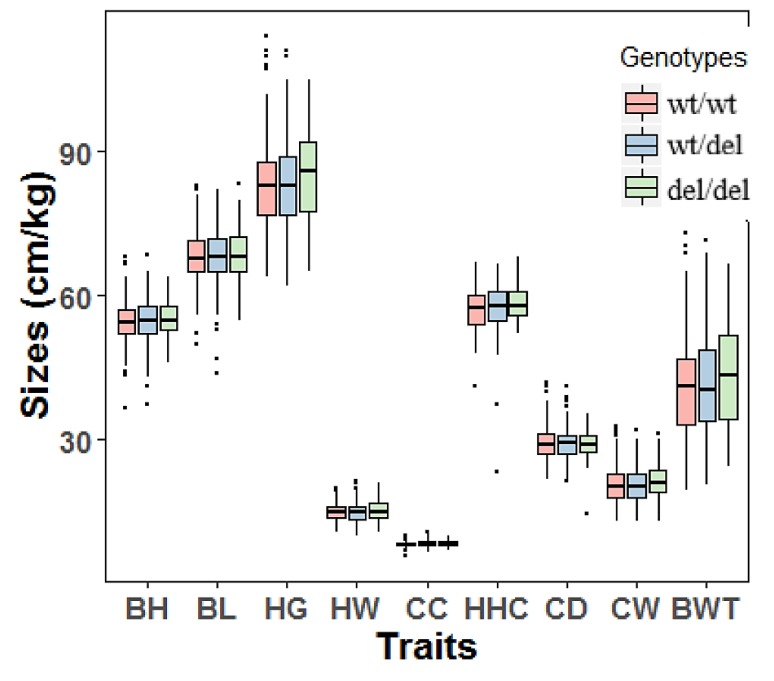
The associations of the 12 bp variation of *PRDM6* and body measurement traits of adult Shaanbei cashmere goats. Wildtype, wt/wt; heterozygote genotype, wt/del, homozygote deletion genotype, del/del. Body height (cm), BH; body length (cm), BL; heart girth (cm), HG; hip-width (cm), HW; cannon circumference (cm), CC; height at hip cross (cm), HHC; chest depth (cm), CD; chest width (cm), CW; body weight (kg), BWT.

The genomic DNA of all 1044 goat individuals, including 567 yearlings (18 months old) and 477 adults (36 months old), was successfully extracted and genotyped. Associations between the 12 bp deletion variation of *PRDM6* and growth traits of yearling SBWC goats were analyzed. For the yearling SBWC goats, analysis revealed that the 12 bp variation was associated with heart girth (*p* = 0.027), cannon circumference (*p* = 0.008), chest depth (*p* = 2.10 × 10^−5^), chest width (*p* = 0.004), body height (*p* = 0.032), body length (*p* = 0.044), and hip-width (*p* = 0.014). The influence of different genotypes on these traits was different (*p* < 0.05) (Table 4 and Figure 4). For the adult SBWC goats, the analysis showed that the 12 bp deletion was not associated with goat growth traits (Table 5 and Figure 5).

## 4. Discussion

Recently, by GWAS studies, the *PRDM6* gene was shown to affect multiple, apparently unrelated inherited traits, including bone density and body mass index; therefore, it was considered as a potentially pleiotropic gene [12,13]. In this study, we wanted to investigate whether *PRDM6* mutations were correlated with body shape and growth-related traits, and we first verified one 12 bp deletion variant located in intron 1 of the goat *PRDM6* gene. For heart girth, cannon circumference, chest depth, and chest width traits of yearling goats, del/del carriers were better than wildtypes, and there were differences between different genotypes and growth traits in yearling goats (Table 4 and Table 5, and Figure 4 and Figure 5). The formation of the skeleton is part of the early development of an organism. Therefore, it is our belief that the *PRDM6* gene affects goat growth in the yearling period, and body sizes might be affected by early skeletal development. This study is consistent with the results of previous studies and demonstrates the validity of our hypothesis. For body height, body length, and hip-width traits, there were no differences between wt/wt genotypes and del/del genotypes. This might be due to the involvement of other genes to regulate these traits. Because multiple genes control goat growth traits, there may be various genes co-regulated in the growth and development of adult goats. Taken together, the 12 bp deletion variation of the *PRDM6* gene plays a vital role in the early growth and development of goats. By the 12 bp mutation, breeders can quickly and effectively select excellent individual goats at an early stage.

PRDM6 belongs to the PRDM family, which possesses C-terminal Krüppel-type zinc finger motifs and the N-terminal PR domain. The PR domain could interact with some essential proteins, such as histone methyltransferase [26,27]. There have been reports showing that PRDM6, as a methyltransferase, was considered to be involved in the histone methylation mechanism in development and reproduction [28,29]. Histone methylation could regulate organic growth and development by epigenetic modification [30,31]. Many studies have proven specific associations between histone methylation and growth and obesity [32,33,34]; therefore, the *PRDM6* gene might affect goat growth and development by being associated with histone methylation. Meanwhile, *PRDM6* is abundant in vascular precursors and inhibits the proliferation and survival program of endothelial cells, so it is critical in maintaining gene expression and cell homeostasis [28,35]. Recently, some reports found that the *PRDM6* gene acted as a potentially pleiotropic gene associated with osteoporosis and obesity [12,13], which illustrates that it may participate in regulating growth and development in various ways. 

**Figure 6:**
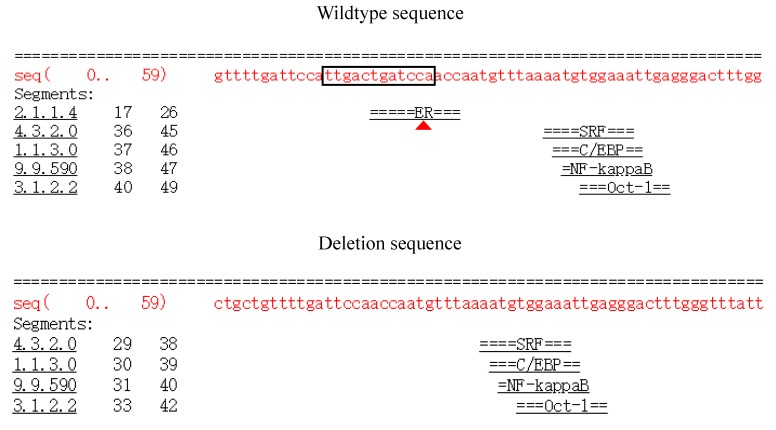
Bioinformatics predicted transcription factor binding sites on the 12 bp mutation sequences of the *PRDM6* gene. The black box highlights the potential transcriptional factor, estrogen receptor (ER), differentially appearing in wildtype sequences. The red triangle represents the differential binding of transcription factors.

Prior studies found that introns had a positive effect on gene expression in various ways; for example, a nucleotide mutation in intron 3 of porcine *IGF2* could affect skeletal muscle by specifically binding to the transcription factor ZBED6 protein [8,36]. Similarly, we wanted to investigate whether this mutation site could affect transcription factor binding differently. According to the AliBaba 2.1 website (http://gene-regulation.com/pub/programs/alibaba2/), compared with the deletion sequence, the 12 bp wild sequence could differently bind to the estrogen receptor (ER) transcription factor (Figure 6). The ER transcription factor has been reported to be essential for bone metabolism via a variety of mechanisms in osteoblasts, osteocytes, and osteoclasts to maintain bone mineral density [37,38]. Thus, we hypothesized that the 12 bp deletion variation might affect the growth process by this transcription factor to regulate *PRDM6* gene expression, but the specific reasons still need further study. Meanwhile, Mandon-Pépin et al. reported that the *GDF9* gene was differentially expressed at the different growth stages of sheep [39]; therefore, we speculated that differences might be due to the differential expression of the *PRDM6* gene in yearling and adult stages.

For the 12 bp deletion mutation, del/del carriers represented better growth-related traits in yearling goats, including heart girth, cannon circumference, chest depth, and chest width traits. There were differences between different genotypes and growth traits in yearling goats, which illustrates that this mutation might exert a large influence in the early growth and development of goats. As we know, the economic value of goats depends on good growth traits as well as the speed of growth. In the process of raising goats, it is of considerable significance to select individuals with a fast growth rate and large body size to improve the economic benefit of the goat industry. In this study, del/del carriers had better body weight and growth traits than wt/wt and wt/del genotype individuals in yearling goats. With the development of modern breeding technology in the future, by selecting this genetic site, breeders can quickly and efficiently carry out early selective breeding of young individuals with better body shape and growth traits. In this way, goats can reach body maturity faster and improve the economic benefit of goat farms, so the 12 bp deletion variation could provide some scientific basis for the efficient and rapid development of the goat industry, and be regarded as an effective molecular marker for goat breeding selection in the early growth stage. 

## 5. Conclusions

A 12 bp deletion mutation within *PRDM6* was identified, and we found that this mutation could affect multiple growth traits of SBWC goats. For heart girth, cannon circumference, chest depth, and chest width traits, compared with wt/wt and wt/del carries, del/del genotypes had the best growth traits in yearling goats. Totally, this 12 bp deletion variation plays a vital role in the early growth and development of goats. It could be used as a useful molecular marker for the early selection of superior individuals in goat breeding.

## Figures and Tables

**Table 1:** Designed primers used for detecting deletion mutations of the goat *PRDM6*.

Primers	Sequences (5’-3’)	Position	RS Number	Length (bp)
*PRDM6*-P1	F: GTTGATGAGGCAGGAGCCTT	upstream	rs656578433	126
	R: GATGCCAGTTTTGTGCCTGG			
*PRDM6*-P2	F: GGATACAGGACAGTGTGGGC	Intron 1	rs651603667	287/275
	R: CAACTCACTGAGCAAGGGGT			

**Table 2:** Mean values of growth traits in yearling and adult Shaanbei white cashmere goats.

Traits	Yearling Population	Sizes	Adult Population	Sizes
heart girth (cm)	73.12	529	83.48	421
cannon circumference (cm)	7.45	530	8.21	421
chest depth (cm)	25.89	530	29.46	421
chest width (cm)	18.94	530	20.99	421
body height (cm)	53.02	567	54.65	470
body length (cm)	62.30	566	68.04	470
Hip-width (cm)	13.03	530	14.99	421
height at hip cross (cm)	55.28	530	57.66	418
body weight (kg)	33.84	161	41.51	452

**Table 3:** Sequence variation frequencies and population indexes for the mutation in Shaanbei white cashmere goats.

Period	Genotypes	Frequency		Ho	He	PIC	χ^2^ (*p* Values)
		Genotypes	Alleles				
Yearling	wt/wt (*n* = 321)	0.566	0.752 (I)	0.627	0.373	0.303	0.002
(18 months old)	wt/del (*n* = 211)	0.372	0.248 (D)				(*p* = 0.967)
	del/del (*n* = 35)	0.062					
Adult	wt/wt (*n* = 220)	0.461	0.667 (I)	0.556	0.444	0.346	2.717
(36 months old)	wt/del (*n* = 196)	0.411	0.333 (D)				(*p* = 0.099)
	del/del (*n* = 61)	0.128					
Sum sample	wt/wt (*n* = 541)	0.518	0.713 (I)	0.591	0.409	0.325	2.326
	wt/del (*n* = 407)	0.390	0.287 (D)				(*p* = 0.127)
	del/del (*n* = 96)	0.092					

Note: ‘*n*’ represents individual numbers. Ho, homozygosity; He, heterozygosity; PIC, polymorphism information content. Wildtype, wt/wt; heterozygote genotype, wt/del, homozygote deletion genotype, del/del.

**Table 4:** Effects of *PRDM6* gene deletion mutations and growth traits of Shaanbei white cashmere goats in the yearling period (mean ± standard errors).

Growth Traits	wt/wt (*n* = 321)	wt/del (*n* = 211)	del/del (*n* = 35)	*p-*Values
HG (cm)	72.27 ^c^ ± 0.37 (*n* = 304)	73.86 ^b^ ± 0.50 (*n* = 195)	76.82 ^a^ ± 1.59 (*n* = 30)	0.027
CC (cm)	7.37 ^C^ ± 0.04 (*n* = 305)	7.54 ^B^ ± 0.05 (*n* = 195)	7.65 ^A^ ± 0.15 (*n* = 30)	0.008
CD (cm)	25.39 ^C^ ± 0.15 (*n* = 305)	26.50 ^B^ ± 0.22 (*n* = 195)	26.98 ^A^ ± 0.54 (*n* = 30)	2.10 × 10^−5^
CW (cm)	18.62 ^C^ ± 0.14 (*n* = 305)	19.27 ^B^ ± 0.19 (*n* = 195)	20.05 ^A^ ± 0.49 (*n* = 30)	0.004
BH (cm)	53.27 ^a^ ± 0.19 (*n* = 321)	52.61 ^b^ ± 0.25 (*n* = 211)	53.28 ^ab^ ± 0.48 (*n* = 35)	0.032
BL (cm)	61.93 ^b^ ± 0.24 (*n* = 321)	62.71 ^a^ ± 0.29 (*n* = 210)	63.31 ^ab^ ± 0.86 (*n* = 35)	0.044
HW (cm)	12.81 ^b^ ± 0.09 (*n* = 305)	13.29 ^a^ ± 0.15 (*n* = 195)	13.63 ^ab^ ± 0.41 (*n* = 30)	0.014
HHC (cm)	55.40 ± 0.20 (*n* = 305)	55.02 ± 0.26 (*n* = 195)	55.86 ± 0.54 (*n* = 30)	0.223
BWT (kg)	33.86 ± 1.08 (*n* = 58)	33.49 ± 0.81 (*n* = 85)	35.39 ± 1.64 (*n* = 18)	0.345

Note: heart girth, HG; cannon circumference, CC; chest depth, CD; chest width, CW; body height, BH; body length, BL; hip-width, HW; height at the hip cross, HHC; body weight, BWT. Wildtype, wt/wt; heterozygote genotype, wt/del, homozygote deletion genotype, del/del. ‘*n*’ represents individual numbers. Cells with different letters (A,B,C; a,b,c) differed significantly (*p* < 0.01; *p* < 0.05).

**Table 5:** Effects of *PRDM6* gene deletion mutations and growth traits of Shaanbei white cashmere goats in the adult period (mean ± standard errors).

Growth Traits	wt/wt (*n* = 220)	wt/del (*n* = 196)	del/del (*n* = 61)	*p-*Values
HG (cm)	83.16 ± 0.64 (*n* = 192)	83.28 ± 0.64 (*n* = 173)	85.21 ± 1.25 (*n* = 56)	0.121
CC (cm)	8.18 ± 0.05 (*n* = 191)	8.24 ± 0.06 (*n* = 173)	8.21 ± 0.10 (*n* = 57)	0.421
CD (cm)	29.51 ± 0.25 (*n* = 193)	29.50 ± 0.25 (*n* = 172)	29.19 ± 0.44 (*n* = 56)	0.531
CW (cm)	21.05 ± 0.28 (*n* = 193)	20.84 ± 0.29 (*n* = 172)	21.27 ± 0.53 (*n* = 56)	0.472
BH (cm)	54.24 ± 0.29 (*n* = 217)	54.95 ± 0.33 (*n* = 193)	55.19 ± 0.49 (*n* = 60)	0.102
BL (cm)	68.05 ± 0.37 (*n* = 217)	67.87 ± 0.43 (*n* = 193)	68.55 ± 0.74 (*n* = 60)	0.423
HW (cm)	14.87 ± 0.13 (*n* = 191)	14.99 ± 0.16 (*n* = 173)	15.39 ± 0.31 (*n* = 57)	0.085
HHC (cm)	57.41 ± 0.30 (*n* = 192)	57.68 ± 0.40 (*n* = 171)	58.45 ± 0.50 (*n* = 55)	0.280
BWT (kg)	40.85 ± 0.74 (*n* = 205)	41.63 ± 0.80 (*n* = 186)	43.39 ± 1.59 (*n* = 61)	0.112

Note: heart girth, HG; cannon circumference, CC; chest depth, CD; chest width, CW; body height, BH; body length, BL; hip-width, HW; height at hip cross, HHC; body weight, BWT. ‘*n*’ represents individual numbers. Wildtype, wt/wt; heterozygote genotype, wt/del, homozygote deletion genotype, del/del.
